# Characterization of the intestinal graft in a swine hypotensive after brain death model^1^

**DOI:** 10.1590/s0102-865020190110000007

**Published:** 2020-01-10

**Authors:** Linlin Li, Ying Gao, Chunlei Lu, Mingxiao Guo

**Affiliations:** I MD, Department of Psychiatry, Linyi Municipal Mental Health Center, Linyi, 276005, China. Acquisition of data, manuscript writing; II MD, Department of General Surgery, Linyi People's Hospital, Xuzhou Medical University, Linyi, 276000, China. Statistics analysis; III MD, Department of General Surgery, Linyi People's Hospital, Xuzhou Medical University, Linyi, 276000, China. Analysis and interpretation of data; IV MD, Department of General Surgery, Linyi People's Hospital, Xuzhou Medical University, Linyi, 276000, China. Conception and design of the study

**Keywords:** Brain Death, Hypotensive, Swine

## Abstract

**Purpose:**

To establish a hypotensive brain death pig model and observe the effects of hypotension on small bowel donors.

**Methods:**

The hypotensive brain death model was produced using the modified intracranial water sac inflation method in ten domestic crossbred pigs. Effects of hypotensive brain death on small bowel tissue morphology were evaluated through changes in intestinal tissue pathology, tight junction protein of the intestinal mucosa and plasma intestinal fatty acid-binding protein (i-FABP) levels. The pathophysiological mechanism was examined based on changes in superior mesenteric artery (SMA) blood flow and systemic hemodynamics.

**Results:**

After model establishment, SMA blood flow, and the mean arterial pressure (MAP) significantly decreased, while heart rate increased rapidly and fluctuated significantly. Small bowel tissue morphology and levels of tight junction protein of the intestinal mucosa showed that after model establishment, small bowel tissue injury was gradually aggravated over time (*P*<0.05). Plasma i-FABP levels significantly increased after brain death (*P*<0.05).

**Conclusions:**

A hypotensive brain death pig model was successfully established using an improved intracranial water sac inflation method. This method offers a possibility of describing the injury mechanisms more clearly during and after brain death.

## Introduction

Intestinal transplantation (ITx) has become an effective treatment for various irreversible intestinal failures. However, the number of patients on the waiting list for small bowel transplantation is larger than the number of available intestinal donors^1^. Currently, most small bowel transplantations are from deceased donors (DDs), while a few come from living donors (LDs)^2^ ^,^ ^3^. Nevertheless, cardiac death donors (DCDs) inevitably suffer a period of warm ischemia before organ donation, which attenuated the graft activity and clinical success of ITx. Therefore, donors after brain death (DBDs) are an ideal donor source. However, a series of pathophysiological changes produced by brain death, especially brain death coupled with circulation instability and extended hypotension, affect the intestinal quality of DBDs. Poor-quality organs significantly affect the survival rate and function of transplant organs^4^ ^,^ ^5^. The pathophysiological mechanism of the effect of hypotensive brain death on donor bowels has not been fully elucidated.

This study established a hypotensive brain death pig model. The utilization of an improved intracranial water sac inflation method is more stable and suitable for the transplantation field. The effects of brain death hypotension on the small bowel donors were observed, on the basis of changes in systemic hemodynamics, intestinal tissue pathology, level of tight junction protein of intestinal mucosa and plasma intestinal fatty acid-binding protein (i-FABP).

## Methods

The animals were treated humanely by use of protocols that were approved by the Institutional Animal Use and Care Committee of Linyi People's Hospital. All procedures were carried out in accordance with “Principles of laboratory animal care” (NIH publication No. 85-23, revised in 1985). Ten domestic crossbred pigs of both sexes, weighing 20-25 kg, were used in the study after a 5- to 7-day acclimatization. Food and water were provided *ad libitum*.

### Experiment design

The baseline data were measured at the end of animal preparation, and then the hypotensive brain death model was induced. After the model was successfully established, the animals were monitored for 12 hours. The animals were sacrificed at the end of the study. T0 to T12 represent 0 – 12 hours after model establishment.

### Experimental preparation

After 24-hr fast with water ad libitum, all animals were premedicated with ketamine (20mg/kg), diazepam (8mg/kg), and atropine (0.06mg/kg) intramuscularly prior to being intubated and anesthetized with propofol (150 mg/kg/hr) and fentanyl (3∼6μg/kg/hr) intravenously. Respiration was supported by a ventilator. Internal jugular vein and carotid arterial catheters were aseptically placed for intravenous access, hemodynamic monitoring, and sample collection. In addition, a 12-lead electrocardiogram was recorded. Laparotomy was performed through a midline incision of the abdomen. Ultrasonic flow probes were inserted around the superior mesenteric artery (SMA). After the bladder puncture catheterization, the abdominal wall was closed. The animal was adjusted to the prone position. A sagittal midline skin incision was performed and the skin was retracted laterally. A craniotomy was performed along the median line of the skull. A 14F Foley catheter was placed in the intracranial epidural space.

### Establishment of a hypotensive brain death model

The catheter was inflated with saline solution at a rate of 0.5mL/min using a peristaltic pump to increase intracranial pressure, and heart rate (HR) and blood pressure were monitored to confirm brain death. The criteria for brain death were as follows: (1) deep coma, excluding reversible factors such as anesthesia and low temperature; (2) absence of oculopupillary and corneal reflexes, repeated twice; (3) loss of spontaneous breathing; (4) no electroencephalographic activity as continuously recorded for 30 minutes; (5) had an atropine test result; (6) disappearance of tic reaction; and (7) showed no changes in characteristics 1 to 6 over a 8-hour period^6^ ^,^ ^7^.

### Monitoring, sampling, and measurements

During the study, the mean arterial pressure (MAP), was maintained at >60 mm Hg; central venous pressure, at 8 to 12mmHg; arterial oxygen saturation, >95%; PaCO2, 35 to 45mm Hg; and body temperature, >36°C as much as possible. The HR, MAP, and SMA blood flow was recorded at baseline and every 2 hours from T0 to T12. A blood sample was extracted from the portal vein at baseline, T0, T4, T8, and T12 to determine the plasma endotoxin and i-FABP levels with an ELISA kit. The intestinal mucosal biopsies were also taken through a midline laparotomy for further analysis at baseline, T4, T8 and T12.

### Histopathological evaluation

Intestinal biopsies were fixed in 10% buffered formaldehyde and embedded in parafin. Histopathology tissue sections, stained with hematoxylin and eosin (H&E), were evaluated by light microscopy. Histological injury of the intestinal samples was quantitatively evaluated according to the scoring system of Park/Chiu^8^. The grades are as follows: 0, normal mucosa; 1, subepithelial Gruenhagen's space (edema) at the apex of the villi; 2, extension of the subepithelial space with moderate epithelial lifting; 3, large subepithelial space and extensive epithelial lifting with occasional denuded villi tips; 4, denuded villi with dilated capillaries; and 5, lamina propria disintegration, hemorrhage, and ulceration.

### Transmission electron microscopy

The pieces of small bowel (2mm×2mm) were washed and fixed with 4% glutaraldehyde for 2 hours and then post-fixed with 1% osmium tetroxide. Tissues were embedded in Epon 812. Thin sections were cut and stained with uranyl acetate and lead citrate and examined with an H-600 (Hitachi, Japan) transmission electron microscope.

### Western blotting of tight junction proteins

Intestinal mucosal biopsies were frozen in liquid nitrogen until further use. Proteins were extracted from intestinal tissues, separated by SDS-PAGE, and then subsequently transferred onto PVDF membranes. The membranes were incubated with primary antibody rabbit anti-ZO-1(1:50, abcam), rabbit anti-occludin (1:250, abcam), or rabbit anti-β-Actin (1:5000, Beijing Zhongshan) over-night at 4°C, followed by exposure to secondary antibody (HRP- anti-rabbit IgG, 1:1000, GenScript) for 2 hours at room temperature. The proteins were visualized with an enhanced chemiluminescent detection system (Thermo) and exposed to X-ray film. Densitometry of the blots was performed using Quantity One 1-D analysis software (Bio-Rad).

### Statistics

All values were expressed as median/range or means ± SD. Data were analyzed with student's t test, by using SPSS 16.0 (SPSS, Inc) software. A *P* value < 0.05 was considered statistically significant.

## Results

Two of the ten animals died from cerebral hemorrhage. The remaining eight animals completed the establishment of a hypotensive brain death model and survived until the end of the study.

### Systemic hemodynamic parameters

**Figure 1:**
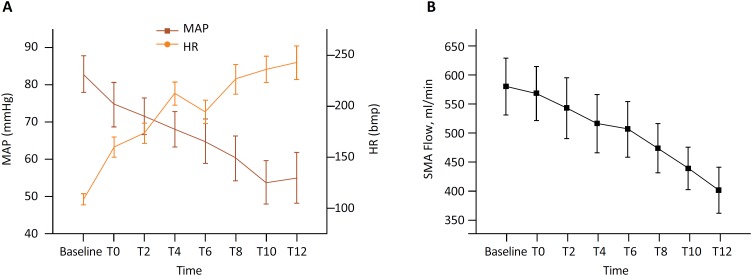
Changes in hemodynamics during the study. Compared with the baseline, *P <.05. **A**, Changes in heart rate and mean arterial pressure during the study; **B**, changes in SMA blood flow during the study.

As shown in Figure 1, HR increased rapidly and fluctuated significantly and the MAP showed a downward trend after T0. Compared with the baseline, the HR at T0 and the subsequent time points increased significantly and the MAP was also significantly reduced (*P*<0.05). After model establishment, the blood flow in the SMA showed a decreasing trend. Compared with the baseline blood flow, the blood flow was significantly reduced at T0 and subsequent time points (*P*<0.05).

### Histopathological evaluation

**Figure 2:**
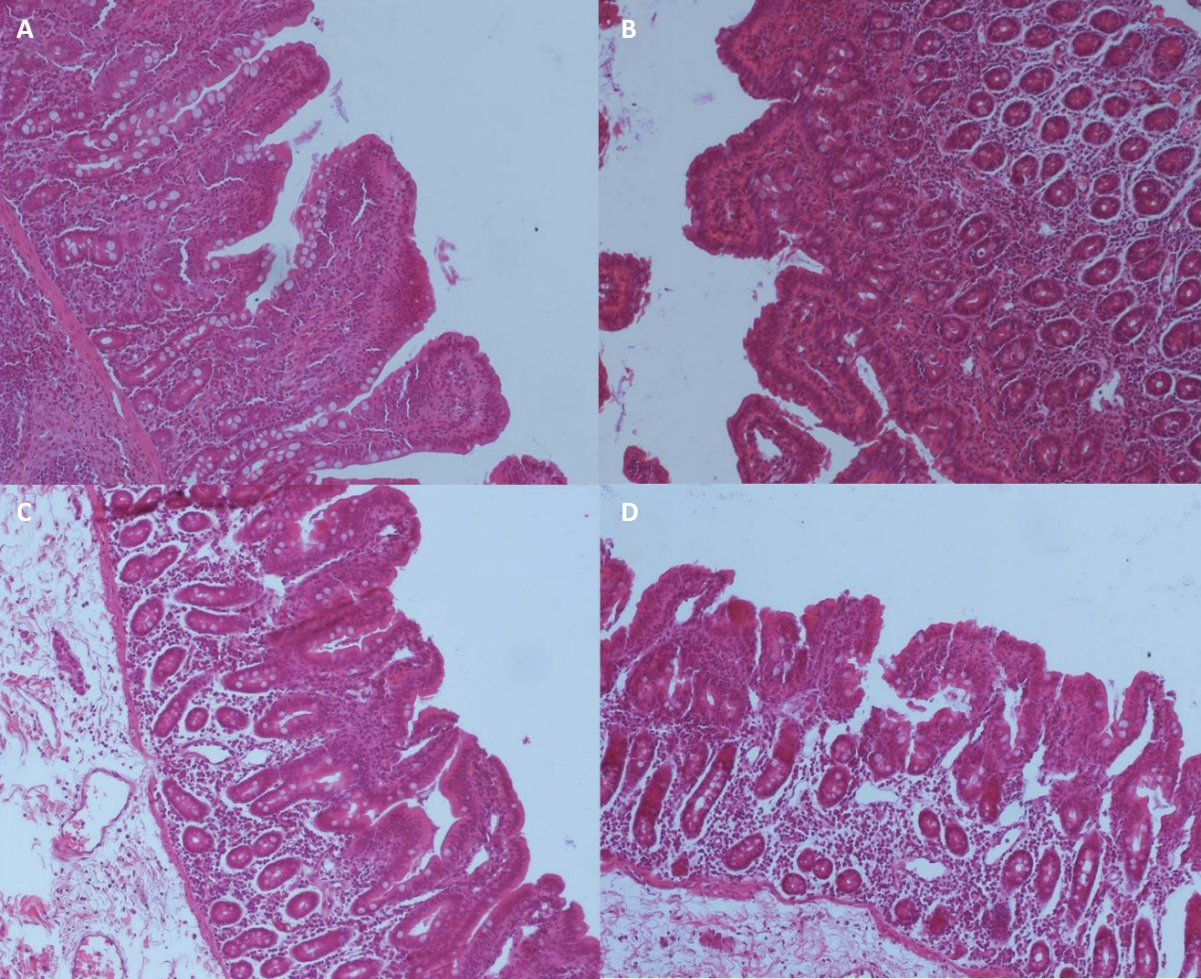
Morphologic changes of small bowel mucosa (HE×100) during the study. **A**, Baseline; **B**, T4; **C**, T8; **D**, T12.

**Figure 3:**
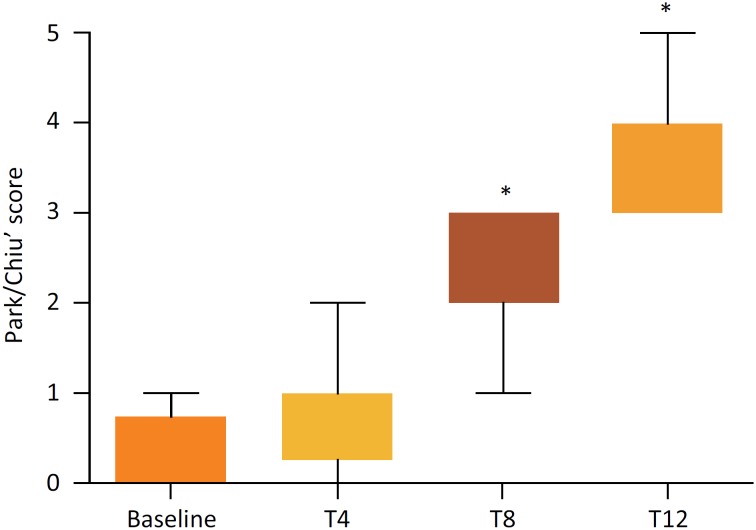
Intestinal injury scores. Compared with the baseline, ^*^ *P*<0.05.

Figure 2A showed integrated villi and compactly arrayed epithelium at baseline. At T4, slight edema and an infiltration of necrotic epithelial cells could be found in the intestinal villi and the gap between epithelial cells slightly increased (Fig. 2B). Based on the Park/Chiu's scoring system, brain death was found to promote intestinal mucosal damage with the prolongation of time (Fig. 3). This result indicated that long term intestinal hypoperfusion may partially intensify intestinal mucosal injury.

### Transmission electron microscopy

**Figure 4:**
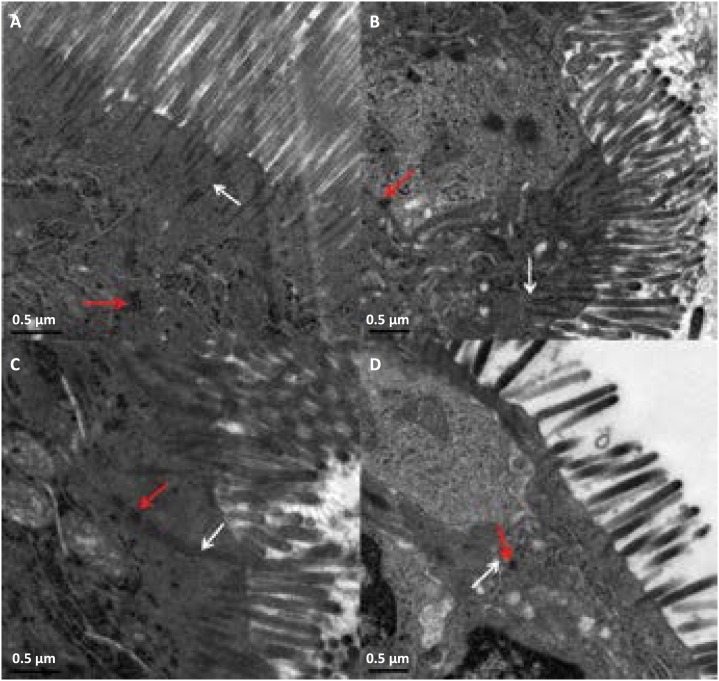
Electron micrograph of intestinal epithelial cells showing tight junction. **A**, Baseline; **B**, T4; **C**, T8; **D**, T12; desmosome (*red arrows*); TJ (*white arrow*).

Tight junctions (TJs) are belt-shaped and expand around the apex of epithelial cells. As shown in Figure 4, the transmission electron microscope showed the microvilli arranged in order, and the TJs appeared as typical membrane fusions with intact TJ structure at the baseline, which can be observed as a linear fusion with high electron density between the adjoining cells. However, a compromised TJ ultrastructure was observed with loss of linear fusion and decreased electron density, and the microvilli were sparse with irregular lengths and arrangements after long term intestinal hypoperfusion.

### Western blotting of ZO-1 and occludin protein

**Figure 5:**
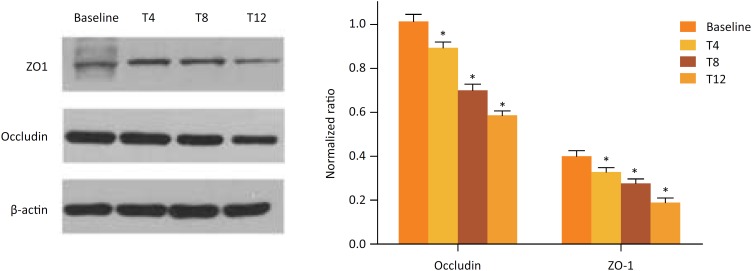
The expression of ZO-1 and occludin proteins in small bowel demonstrated by western blot analysis. Levels of the indicated protein at the baseline, T4, T8 and T12.

Western blotting analysis showed that there was a statistically significant decrease in occludin and ZO-1 expression post brain death when compared to baseline (*P*<0.05) (Fig. 5).

### Serum concentration of i-FABP

**Figure 6:**
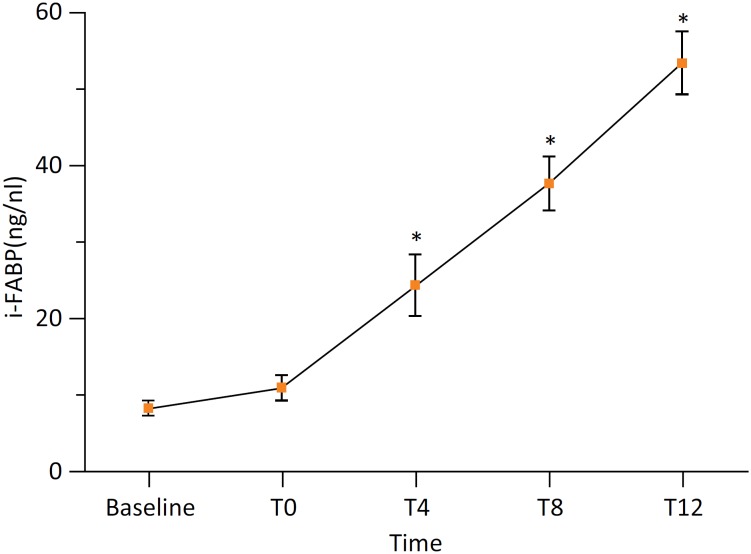
Intestinal permeability and damage were determined with serum i-FABP. Compared with the baseline (^*^ *P*<0.05).

Serum i-FABP levels showed elevated trends after model establishment. Compared with baseline values, serum i-FABP levels were significantly increased at 4, 8, and 12 hours after brain death (*P*<0.05) (Fig. 6).

## Discussion

Intestinal transplantation is an effective treatment for various irreversible intestinal failures^1^. DBDs are one of the sources of organs for intestinal transplantation in most countries^3^. However, despite progressive improvement in immunosuppression, both the short- and long-term success rates for transplantation remain substantially inferior to solid organ, which may be related to the sensitivity of small intestine to ischemia. Therefore, establishment of a stable, reproducible, and hypotensive animal model of DBDs is important to understand the effect of brain death on intestinal donor quality.

The most recent application is the classic epidural water sac inflation method^9^ ^,^ ^10^. The rate and amount of water injected is the key to this modeling method, which have reported differences in previous literatures, and caused obvious differences among the established brain death animal models. Therefore, these models cannot accurately assess the physiological and biochemical changes of DBDs^11^ ^,^ ^12^. We modified the classic method by slowly discontinuously injecting using a peristaltic pump to increase intracranial pressure. This can prevent a very fast and/or high increase in intracranial pressure, which can cause unnecessary damage to the brain, and also an excessive fluctuation of intracranial pressure, which can cause excessive fluctuations of the cycle and cardiac functions. In this study, the hypotensive brain death model was successfully established in all surviving animals, and monitoring indicators such as HR, MAP, and SMA blood flow showed that the induction process was stable, with the MAP fluctuated at 60mmHg during the study.

Brain death often engenders hemodynamic changes, hormonal alterations, blood coagulation factor consumption, lung tissue changes, hypothermia, and electrolyte disturbance^13^. Thus, changes invariably influence intestinal morphology and functions. The morphologic integrity of the small bowel graft is paramount to preventing bacterial translocation and subsequent infectious complications of the recipients. Koudstaal *et al*.^14^ ^,^ ^15^ found that intestinal mucosal injury was clearly present 1-hour after brain death in rats compared to controls. Nevertheless, we found no significant change in small bowel tissue pathology observed 4 hours after hypotensive brain death, and that the reversible damage increased gradually 4 to 12 hours after hypotensive brain death. This may be related to the differences of species. Söfteland *et al*.^16^ confirmed that porcine intestines have a slower development of tissue injury compared to human intestines, while rat intestines appear to have a faster injury development. Similar to the changed pathology, the transmission electron microscope also found that the TJ ultrastructure and microvilli were significantly altered after a period of hypotension. The explanation for these remarkable interspecies differences remains unknown. However, the practical consequence of this finding is that ischemic studies using pig small bowels would need a longer time than those using rats, to attain a significant amount of tissue injury. The current results further confirm that pigs have important anatomical and physiological similarities to humans including comparable digestive function and intestinal structure and physiology making this species a useful animal for preclinical research^17^.

In contrast with the mild histologic changes, we also found some early molecular changes involving the TJs. TJs are the most apical component of intercellular junction complexes, thus functioning as major determinants of intestinal epithelial barrier function. Disruption of the tight junctions can cause an increased permeability and leakiness. Oltean *et al*.^18^ found that expression of all proteins within the junctional complex was subjected to various durations of cold storage. ZO-1, tricellulin, and occludin were significantly decreased after 8 hour of cold storage and continuously worsened, while Claudin-3, Claudin-4 and E-cadherin expression remained relatively unaltered during cold storage^18^. Therefore, the changes of ZO-1 and occludin could reflect the condition of TJs and intestinal mucosa barrier. The current study showed that the expression of ZO-1 and occludin was significantly reduced after model establishment and subsequent time points, which was similar to the results of transmission electron microscopy and pathological results. In addition, plasma i-FABP, which is a sensitive biochemical marker of early gut mucosal injury^19^ ^,^ ^20^, was also evaluated in our study. Normally, the content of i-FABP in serum is very low, which is difficult to detect. However, i-FABP can be detected in plasma and urine after damage of cells. Increased i-FABP concentrations after model establishment indicated intestinal dysfunction in brain-dead pigs.

Brain death is a complex situation with an enormous number of variables affecting donor organs. Golling *et al*.^21^ found in a pig brain death model that systemic perfusion parameters, intestinal ischemia, and oxidative stress were affected by the hemodynamic status. Van Der Hoeven *et al*.^22^ found that maintaining normal blood pressure after brain death could reduce the damage of the intestinal mucosa, but it could not completely prevent other functional disorders associated with brain death. McCuskey *et al*.^23^ found that hypotension could lead to obvious gastrointestinal ischemia and was associated with intestinal mucosal permeability increase and endotoxin displacement. In this study, SMA blood flow monitoring showed that the gastrointestinal tract presented a low perfusion state under hypotensive brain death. Under the state of brain death, hemodynamic abnormalities and low perfusion of the small bowel may impair the donor bowel through direct and/or indirect pathways.

## Conclusions

A hypotensive brain death pig model was successfully established using an improved intracranial water sac inflation method. Gut injury was found to be progressively aggravated under a state of hypotensive brain death. Therefore, it offers the possibility to describe the injury mechanisms during and after BD, allowing evaluation of new strategies to ameliorate intestinal quality and patient survival after intestinal transplantation.
